# Adaptive Lévy Processes and Area-Restricted Search in Human Foraging

**DOI:** 10.1371/journal.pone.0060488

**Published:** 2013-04-05

**Authors:** Thomas T. Hills, Christopher Kalff, Jan M. Wiener

**Affiliations:** 1 Department of Psychology, University of Warwick, Coventry, United Kingdom; 2 Center for Cognitive Science, University of Freiburg, Freiburg, Germany; 3 Psychology Research Centre, Bournemouth University, Bournemouth, United Kingdom; University of Zaragoza, Spain

## Abstract

A considerable amount of research has claimed that animals’ foraging behaviors display movement lengths with power-law distributed tails, characteristic of Lévy flights and Lévy walks. Though these claims have recently come into question, the proposal that many animals forage using Lévy processes nonetheless remains. A Lévy process does not consider when or where resources are encountered, and samples movement lengths independently of past experience. However, Lévy processes too have come into question based on the observation that in patchy resource environments resource-sensitive foraging strategies, like area-restricted search, perform better than Lévy flights yet can still generate heavy-tailed distributions of movement lengths. To investigate these questions further, we tracked humans as they searched for hidden resources in an open-field virtual environment, with either patchy or dispersed resource distributions. Supporting previous research, for both conditions logarithmic binning methods were consistent with Lévy flights and rank-frequency methods–comparing alternative distributions using maximum likelihood methods–showed the strongest support for bounded power-law distributions (truncated Lévy flights). However, goodness-of-fit tests found that even bounded power-law distributions only accurately characterized movement behavior for 4 (out of 32) participants. Moreover, paths in the patchy environment (but not the dispersed environment) showed a transition to intensive search following resource encounters, characteristic of area-restricted search. Transferring paths between environments revealed that paths generated in the patchy environment were adapted to that environment. Our results suggest that though power-law distributions do not accurately reflect human search, Lévy processes may still describe movement in dispersed environments, but not in patchy environments–where search was area-restricted. Furthermore, our results indicate that search strategies cannot be inferred without knowing how organisms respond to resources–as both patched and dispersed conditions led to similar Lévy-like movement distributions.

## Introduction

Numerous species have been proposed to display power-law distributed movement patterns when foraging [1]–[5]. Power-law distributed movement patterns are superdiffusive, with straight-line movement length, *l*, having probability distribution function 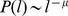, with 1<*μ*<3. A common interpretation of power-law distributed movements is that they represent the outcome of Lévy walks (with probabilities based on duration) or Lévy flights (with probabilities based on distance traveled) [2], [5], [6]. When velocities are constant, we can consider these two synonymous, as we do here (and simply use the term Lévy flights). Both refer to scale-free random walks in which run duration or movement lengths are independently drawn from a probability distribution with a heavy power-law tail. Though the power-law distribution of movement lengths for many organisms has come into question [7], the processes which create animal paths are potentially still Lévy processes. We define a Lévy process with respect to foraging as a stochastic process in which increments are independently drawn and statistically identical for non-overlapping portions of the path [8]. Examples of Lévy processes are Brownian motion, Lévy flights, Lévy walks, and Poisson processes. Because the underlying movement distributions do not change in response to resource encounters, Lévy processes imply that organisms do not use information about recent resource encounters to localize search in space.

In contrast to Lévy processes, patterns of extensive and intensive foraging in response to resource absence or presence, respectively, have also been widely observed across species [9]–[11]. This pattern of movement is called area-restricted (or area-concentrated) search. Area-restricted search requires memory in order to create local intensive searching around locations where resources have been found in the past. Moreover, area-restricted search is capable of generating distributions of movement lengths with heavy-tailed power-law distributions [12], [13]. Though some work has been interpreted as suggesting that Lévy flights are optimal foraging strategies [14], these were not compared with alternative strategies like area-restricted search. Comparisons of these foraging strategies in destructive foraging environments–where resources are not replaced–have found that when resource locations are spatially uncorrelated (distributed independently), ballistic foraging strategies are optimal, whereas when resource locations are spatially auto-correlated (distributed in clusters), then area-restricted search strategies are optimal [12]–[16].

Historically, the methodological difficulties associated with determining what generates a power-law distribution have led to considerable debate over which animals, if any, actually use Lévy processes [4], [6], [7], [12], [17], [18]. In part, this argument has tried to address whether animals do Lévy flights by focusing on the statistical methodology used to identify the underlying distributions [4], [7], [16], [17]. Still others have investigated behavioral mechanisms that can generate such distributions [12], [13], [19], [20]. Here we take a different approach by focusing on the fact that a Lévy process samples from the same movement length distribution without regard to resource encounters, whereas area-restricted search strategies are processes that sample from different distributions depending on the time passage since last resource encounter [21]–[23]. Thus, our approach to identifying the underlying data generating process requires knowing exactly where resources are and how behavior changes in response to encountering them.

In the present study we focus on human foraging. Consistent with what has been shown for other animals, several studies have attempted to show that human movement patterns may be Lévy flights [2], [24]–[26]. However, other studies have suggested that humans do not use Lévy flights, because–using maximum likelihood methods and goodness-of-fit tests–the observed distributions were found not to follow power-law distributions [7]. No previous studies have investigated the broader theoretical question of Lévy processes in humans, nor have previous studies investigated how human search may respond adaptively to the correlational structure of resource distributions.

Here we present an analysis of human foraging in a virtual environment, resembling a large open field. Using both clustered (patchy) and dispersed (non-patchy) resource distributions, we tracked individual search trajectories and resource encounters and asked to what extent paths were adapted to their specific sequence of resource encounters. Our aim was to determine how the movement lengths of human search trajectories are distributed and to address under what circumstances these distributions may represent Lévy processes or area-restricted search.

## Methods

**Figure 1:**
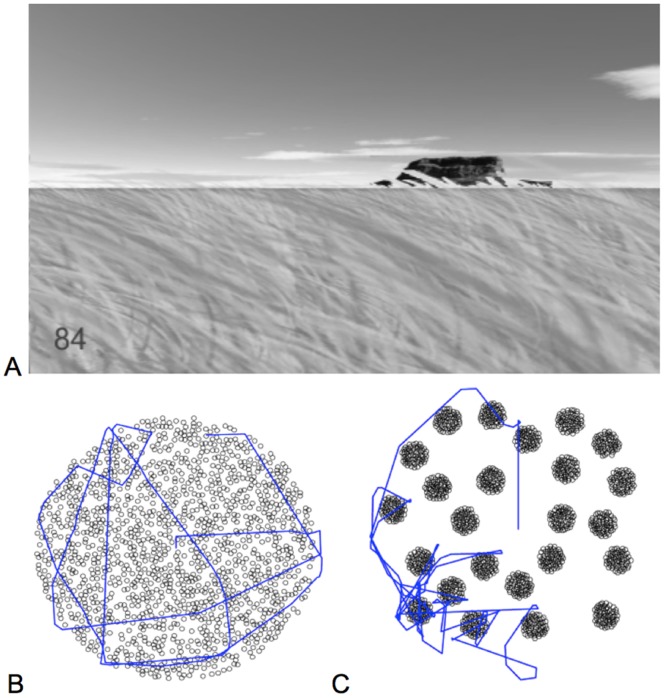
The virtual foraging environment, resource distributions, and representative paths. **A.** Participants’ perspective during the task. One of the global landmarks (a mountain) is visible in the distance. The number in the lower left hand corner is the number of resources collected so far. **B.** The resource distribution in the dispersed environment with a path generated by one participant. **C.** The resource distribution in the patchy environment with a path generated by one participant.

Participants (*n* = 32) searched in a circular virtual arena that contained hidden targets. The environment consisted of a textured ground plane resembling a large meadow and was surrounded by a fence, with large distal landmarks (e.g., mountains) to provide global orientation cues. There were no local cues, such as depressed grass, to signal where participants had been (Fig. 1A). Targets were either uniformly distributed (dispersed condition, Fig. 1B) or organized in patches (patched condition, Fig. 1C) in a between subjects design. People searched the virtual circular meadow (110m radius) displayed on three computer screens, representing 180*°* field of view. They did this using the arrow keys, which allowed them to either move forward or turn, but not both at the same time. The distance between two turns was defined as a movement length. 1440 items were randomly located: in the dispersed condition locations were independently and uniformly determined; in the patched condition, the centers of 24 patches were uniformly assigned, but non-overlapping, and 60 items were randomly located within 8.65 meters of the patch center. Participants heard a tone when they encountered an item (detected at a distance of 0.75 meters) and were required to search for and collect 90 items. The participants were randomly assigned to the two conditions, told to search for 90 items, with the search repeated 5 times for each participant. Participants were not told about the resource distribution. However, participants appear to have learned this rapidly, because behavior did not substantially vary over the 5 repeated foraging trials. We therefore report our analyses on the aggregated individual data over the 5 trails.

Statistical analyses used standard likelihood methods and Akaike weights to compare four statistical models: unbounded power-law, bounded power-law, unbounded exponential, and bounded exponential. Methods and code can be found in previous work [7], [27]. For reference with past literature supporting Lévy flights, we also present results based on logarithmic binning [18], [28]. In order to evaluate whether or not movements were independent of recent resource encounters, we compared observed turning with baseline turning following resource encounters. Baseline turning was measured by selecting random locations along the recorded trajectories and calculating the turning response as a function of the distance after these random locations (“Random dispersed” and “Random patched”). To establish whether or not paths were adapted to their environments, we compared paths across environments; paths observed in one resource distribution were simulated 100 times in the alternative resource distribution by rotating them using a uniform random sampling of the initial heading around 360*°*.

## Results

**Figure 2:**
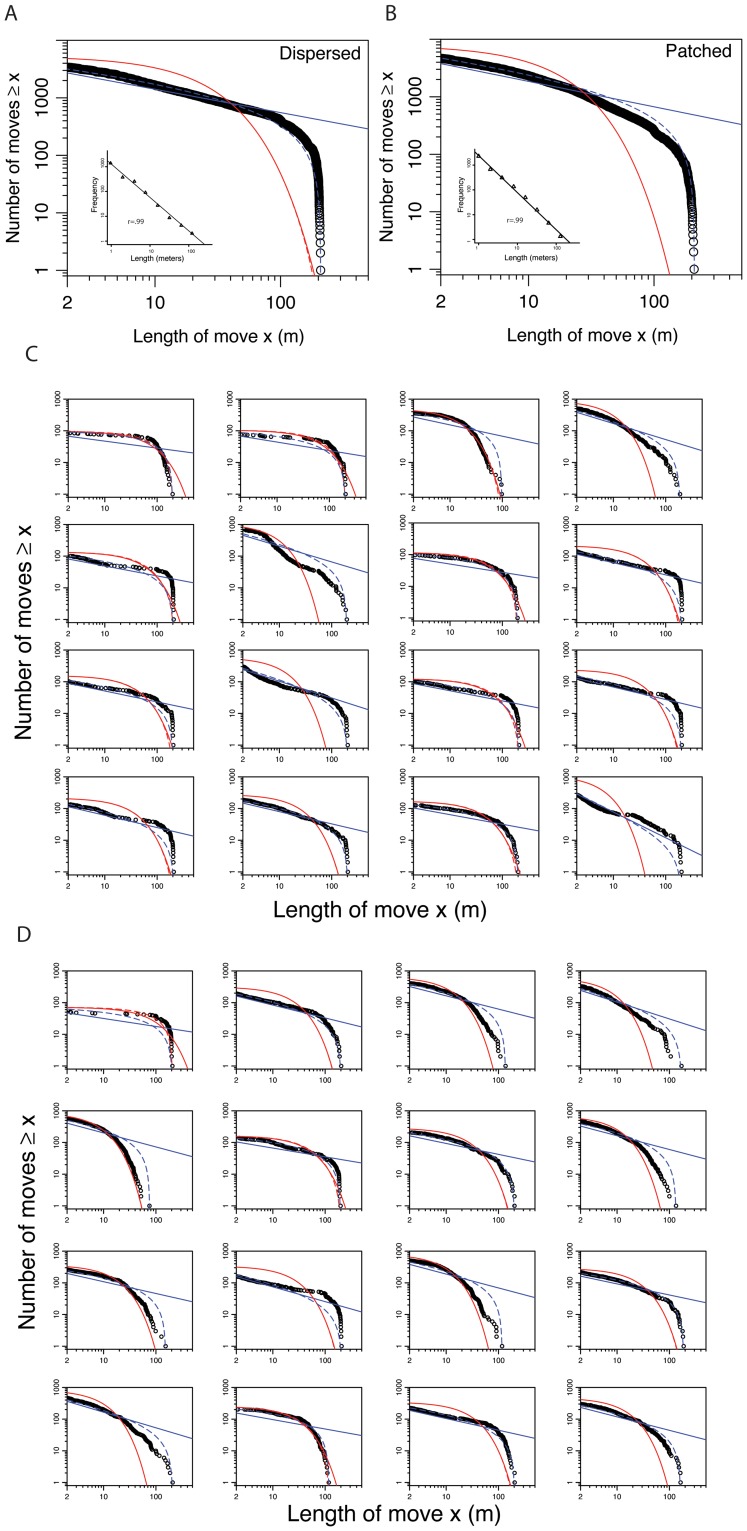
Rank/frequency plots of aggregated and individual data along with model fits on logarithmic axes. Black circles are movement lengths ≥ *x*. The four model fits are power-law (blue-straight line), bounded power-law (curved blue-dashed line), unbounded exponential (curved red line), and bounded exponential (curved red-dashed line). **A.** The aggregated data for the dispersed condition. The inset shows the results of logarithmic binning with best fitting power-law. **B.** The aggregated data for the patched condition. The inset shows the results of logarithmic binning with best fitting power-law. **C.** Data for each individual in the dispersed condition. **D.** Data for each individual in the patched condition.

Figure 2 presents rank/frequency plots of the data and the model fitting for the aggregated data from each condition and for each individual separately. Data are presented on logarithmic axes because a power-law distribution appears as a straight line on these axes. For the aggregated models (Fig. 2A, B), only the bounded power-law appears to fit the data with any degree of precision. The unbounded power-law overestimates the size of longer moves and the exponential fits underestimate these longer moves. For the individual data, model fits vary widely (Fig. 2C, D), with few individuals appearing to be well described by any statistical model.

Before we discuss the statistical tests associated with these distributions, we first present the results of logarithmic binning. For reference with previous literature [1], [5], [14], [16], [18], [28]–[30], the insets of Figure 2A and 2B present the data using logarithmic binning methods. The slope for the patched condition was *μ = *−1.45±0.40 and for the dispersed condition was *μ = *−1.23±0.31. These were not statistically different (*P>*.05). Though necessary for an interpretation of Lévy flights, these results are far from sufficient. Moreover, the method of logarithmic binning has come under attack for multiple reasons and fails to compare alternative hypotheses [7], [27].

Our statistical analyses thus used standard likelihood methods and Akaike weights to compare four statistical models based on the rank/frequency plots: unbounded power-law, bounded power-law, unbounded exponential, and bounded exponential. Table 1 presents the analyses based on aggregated data, providing the evidence ratios for the different models–which represent the Akaike weight of a model divided by the best fitting Akaike weight, such that the best fitting model has a value of 1.0 and other models have values >1.0. Table 2 presents the analyses based on individual data, showing the proportion of participants best fit by each model. For both aggregated and individual data, the bounded power-law model (truncated Lévy flight) was the best fitting model in all cases. We used a G-test (likelihood ratio test) to compare the bounded power-law with the data, with the null hypothesis that the data are consistent with this model [7]. Both aggregated data sets failed the goodness of fit test (Table 1) and all but two individuals in each condition failed the goodness of fit test (Table 2). This indicates that even the truncated Lévy flight–despite it being the best of the models we tested–still appears to poorly characterize human behavior.

**Table 1:** Model comparisons for aggregated data.

	Evidence ratios				Best model’sgoodness of fit	
Aggregated	PL	Exp	PLB	ExpB	*n*	*P*
Dispersed	>10^30^	>10^30^	**1.0**	>10^30^	5210	0
Patched	>10^30^	>10^30^	**1.0**	>10^30^	7688	0

Note: PL = power law, Exp = unbounded exponential, PLB = bounded power-law, ExpB = bounded exponential.

**Table 2:** Model comparisons for individual data.

	Proportion best fit by each model				Proportion with *P>*.05 for best model
Individual	PL	Exp	PLB	ExpB	
Dispersed	0	0	**1.0**	0	.125
Patched	0	0	**1.0**	0	.125

Note: PL = power law, Exp = unbounded exponential, PLB = bounded power-law, ExpB = bounded exponential.

In addition to the model fitting, we also found that the two conditions did not differ in mean movement length (*M_dispersed_ = *43.2, *M_patched_ = *31.1, *t(30) = *1.35, *P = *.19, two-tailed t-test), mean absolute turning angle (*M = *53.8, *M = *52.5, *t(30) = *0.19, *P = *.85, two-tailed t-test), or mean *μ* associated with the best fitting bounded power-law model (*M_dispersed_* = 1.06, *M_patched_* = 1.16, *t*(30) = −1.11, *P = *.27, two-tailed t-test). These results lend themselves to two conclusions. Foremost, despite a strong apparent fit to power-law distributions when using logarithmic binning, the movement distributions are not well described by power-law distributions and therefore fail to meet a basic requirement of Lévy flights. Second, the two conditions do not appear to be significantly different from one another based on movement distributions alone, and are therefore potentially consistent with a common underlying search strategy (but see below).

The first conclusion is likely to come under some criticism. Only bounded power-laws are meaningful in natural systems, because “all power laws in nature have upper and lower cutoffs” (p. 41, [16]). Thus, realistically, we can expect true Lévy-like behaviors to be best characterized by truncated Lévy flights, especially if foragers stop when encountering items. Failures to fit bounded power-law distributions may simply reflect improper bounds, which may in this case be a function of, for example, human reaction times or different cognitive processes being used over very short or very long movement intervals. Despite failing the goodness of fit tests, because our data are statistically most consistent with truncated Lévy flights this may lead some readers to infer that the processes underlying the movement are indeed Lévy processes. But this is an unfounded inference. Even if the distributions were bounded power-law distributions, different behavioral processes (besides Lévy processes) can generate bounded power-law distributions. As noted in previous literature, inferences based on distributions alone are insufficient evidence to infer Lévy flights [12], [13]. Ruling out such alternative explanations requires an analysis of movement based on where and when resources were encountered.

**Figure 3:**
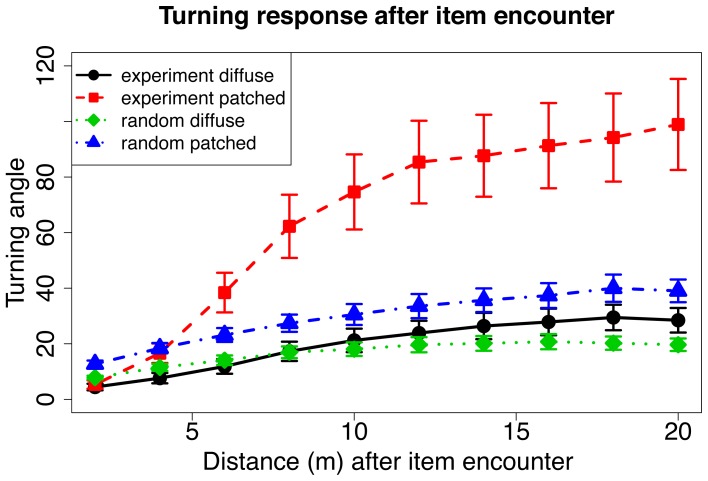
Turning angle as a function of distance after item encounter for the empirical data (“Experiment dispersed” and “Experiment patched”) and for random locations along the trajectories (“Random dispersed” and “Random patched”). Participants in the patched condition significantly increased turning in response to resource encounters relative to both the dispersed condition (*F(1,30) = *5.31, *P = *.03, repeated measures analysis of variance) and ‘random’ baseline turning (*F(1,15) = *5.71, *P* = .03, repeated measures analysis of variance). Turning angles in the dispersed condition were not different from the ‘random’ baseline turning (*F(1,15) = *1.68, *P = *.21, repeated measures analysis of variance). Data show mean±sem.

To address this issue, we compared turning angles following resource encounters for both patched and dispersed conditions with a baseline reference class of turning angles evaluated at random points along participants’ paths (Fig. 3). If individuals increase their turning angles above the baseline in response to encountering a resource item, this suggests that movement lengths are not independently sampled, but reflect the participant’s initiating intensive foraging. Indeed, following a resource encounter turning angles in the patchy environment were sharper than in the dispersed condition (*M_patched_ = 65.47°, M_dispersed_ = 19.85°*) and sharper than turning angles taken relative to random points along the path (*M_random patched_ = 29.79°*). Thus, for the patched condition, the results support a transition to an intensive search following resource encounters, confirming area-restricted search. The observed turning angles for the dispersed condition were not different from their random reference class (*M_dispersed_ = 19.85°,M_dispersed_random_ = 16.89°*), indicating insensitivity to resource encounters and consistent with a Lévy-flight-like process (possibly a truncated Lévy flight).

**Figure 4:**
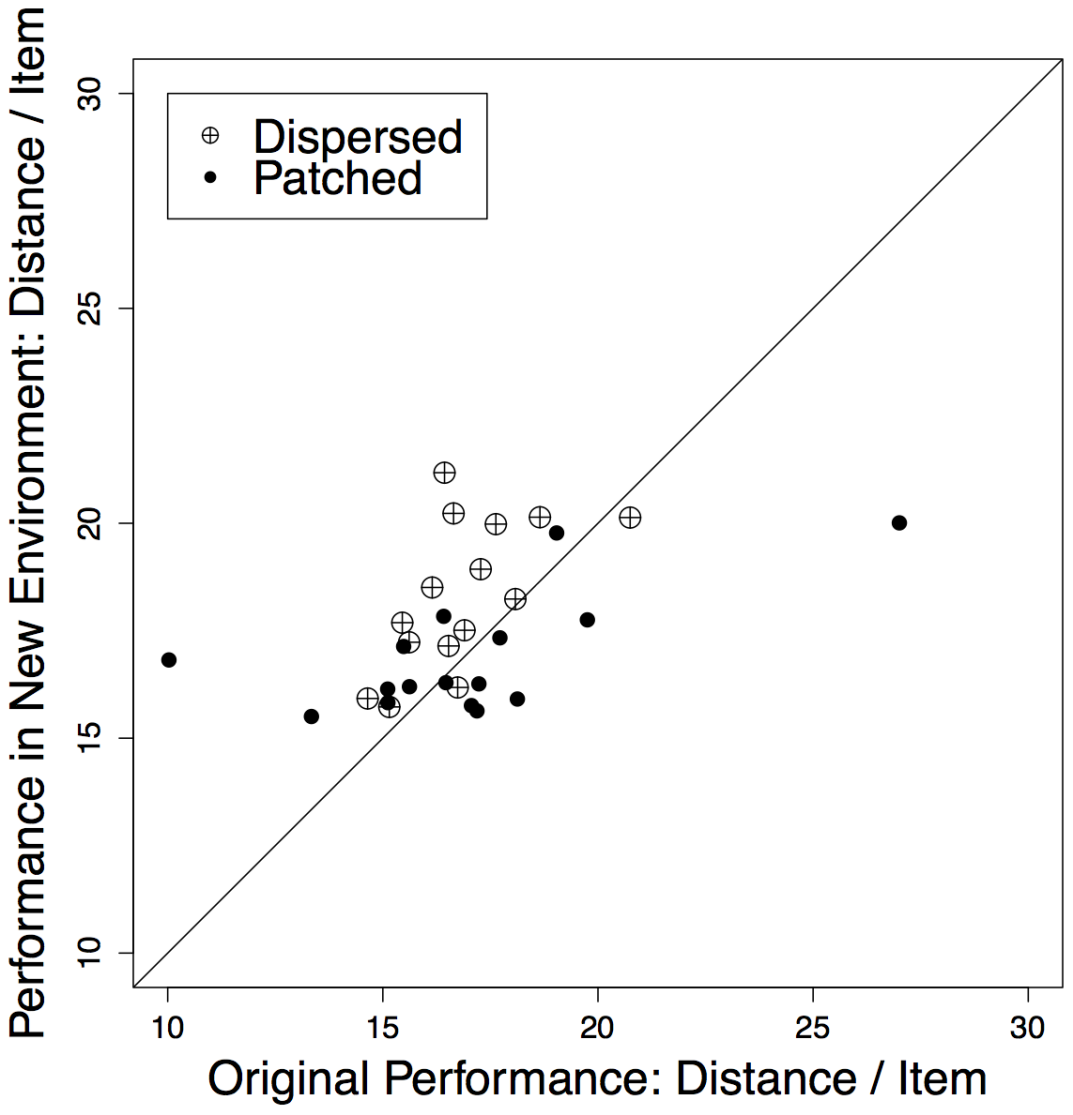
Comparing path performance across environments. We compared path performance by randomly simulating paths from the alternative environment using 100 simulated versions of each observed path in the alternative resource distribution. Paths from the patched condition simulated in the dispersed environment performed as well as dispersed paths in the dispersed environment (*t*(15) = 0.05, *P* = .97, two-tailed t-test). However, paths from the dispersed environment simulated in the patchy environment were outperformed by the original paths from the patchy environment (*t*(14) = −3.91, *P = *.002, two-tailed t-test).

Was the area-restricted search in the patched condition associated with improved performance, as proposed in previous literature [12], [13], [16]? To establish whether increased turning following resource encounters was an adaptive change in search strategy, we asked how the paths produced in one environment would have performed had they been observed in the other environment (Fig. 4). Paths transferred from the dispersed environment to the patchy environment performed worse than paths originally generated in the patchy environment (observed - new: *M_dispersed_* = −1.47, *SD = *1.46). However, paths transferred from the patchy environment to the dispersed environment did not perform differently than the original dispersed paths (*M*_patched_ = −0.31, *SD = *2.84). This is consistent with previous theoretical claims and demonstrates empirically that in patchy environments paths adapted to the spatially auto-correlated structure of the resource environment–responding to resource encounters with intensive search–are more efficient than a putative Lévy-flight-like process. However, in the spatially uncorrelated resource environment, information about resource locations was not provided by resource encounters and participants could efficiently utilize a random Lévy-like process.

## Discussion

The present work follows Benhamou [12] in suggesting that the test for a Lévy flight requires two components: 1) an analysis of path distribution, and 2) evidence that the path is intrinsically generated and not a result of external resource encounters. Our results demonstrate that these two criteria are possibly met for humans foraging in dispersed, spatially uncorrelated resource environments–where we found movement lengths most consistent with a bounded power-law, though these failed the goodness of fit tests. These paths also showed no sensitivity to resource encounters, suggesting they may be consistent with Lévy processes. On the other hand, humans exposed to spatially auto-correlated resource environments, with resources distributed in patches, showed similarly distributed movement lengths but adapted their search to the structure of the environment by responding to resource encounters with increased turning, characteristic of area-restricted search.

The putative claim for Lévy flights in diverse categories of living organisms–ranging from T cells to hunter-gatherer foraging camps [1], [2], [5], [6], [31]–raises fundamental questions about the underlying generative processes driving these behaviors and the optimality of the resulting search. Our results offer potential inroads to future studies, as well as providing grounds for alternative explanations. In particular, putative Lévy flights may adapt to the resource structure of their environment by a change in the characteristic scale of their movement length distribution [6], movement distributions similar to bounded power-law distributions and possibly changes in movement length distributions may further arise as a result of adaptive changes in behavioral responses to encounters with resources. Because of the similar nature of the two movement length distributions in our two conditions, our results further warn against inferring behavior based on curve fitting.

In addition, when behavioral ecologists have investigated how animals respond to resources, area-restricted search has been observed in animals across the metazoan lineage (e.g., vertebrates and invertebrates) and typically involves similar neuromolecular mechanisms [32]. A common hypothesis when observing both shared traits and shared mechanisms is that the trait existed in an ancestor common to the different species under study. In the case of metazoans, this species would have existed approximately 6 to 7 hundred million years ago. This indicates that area-restricted search is likely to be an extremely common strategy for localizing search around patchy resources in space and should, at the least, represent an alternative hypothesis for comparison in future studies of Lévy-like movement patterns.

Finally, we note that the observed relationship between Lévy-flight-like processes and area-restricted search, in a single animal (i.e., humans), provides a foothold for further investigating the behavioral and neuromolecular mechanisms driving power-law distributed behavior across a wide range of species and environments [3], [12], [29], [33], [34]. This is in part because the neuromolecular mechanisms underlying behavioral changes in response to environmental rewards are well studied [10], [35]–[37], which allows us to pose new questions for our understanding of the physiological and evolutionary origins of power-law distributed behavior patterns, specifically in terms of how they may be a response to resources.
